# The Risk of Oxygen during Cardiac Surgery (ROCS) trial: study protocol for a randomized clinical trial

**DOI:** 10.1186/s13063-017-2021-5

**Published:** 2017-06-26

**Authors:** Marcos G. Lopez, Mias Pretorius, Matthew S. Shotwell, Robert Deegan, Susan S. Eagle, Jeremy M. Bennett, Bantayehu Sileshi, Yafen Liang, Brian J. Gelfand, Adam J. Kingeter, Kara K. Siegrist, Frederick W. Lombard, Tiffany M. Richburg, Dane A. Fornero, Andrew D. Shaw, Antonio Hernandez, Frederic T. Billings

**Affiliations:** 1 Division of Anesthesiology Critical Care Medicine, Department of Anesthesiology, Vanderbilt University Medical Center, 1211 21st Avenue South, Suite 526, Nashville, TN 37212 USA; 2 Division of Cardiothoracic Anesthesiology, Department of Anesthesiology, Vanderbilt University Medical Center, 1211 21st Avenue South, Suite 526, Nashville, TN 37212 USA; 3 Department of Biostatistics, Vanderbilt University Medical Center, Nashville, TN USA; 4 Cardiovascular Perfusion Technology Program, Vanderbilt University Medical Center, Nashville, TN USA

**Keywords:** Normoxia, Physiologic oxygenation, Hyperoxia, Hyper-oxygenation, Oxidative stress, Reactive oxygen species, Acute kidney injury, Endothelial dysfunction, Delirium, Isoprostanes, Isofurans, Cardiac surgery, Clinical trial

## Abstract

**Background:**

Anesthesiologists administer excess supplemental oxygen (hyper-oxygenation) to patients during surgery to avoid hypoxia. Hyper-oxygenation, however, may increase the generation of reactive oxygen species and cause oxidative damage. In cardiac surgery, increased oxidative damage has been associated with postoperative kidney and brain injury. We hypothesize that maintenance of normoxia during cardiac surgery (physiologic oxygenation) decreases kidney injury and oxidative damage compared to hyper-oxygenation.

**Methods/design:**

The Risk of Oxygen during Cardiac Surgery (ROCS) trial will randomly assign 200 cardiac surgery patients to receive physiologic oxygenation, defined as the lowest fraction of inspired oxygen (FIO_2_) necessary to maintain an arterial hemoglobin saturation of 95 to 97%, or hyper-oxygenation (FIO_2_ = 1.0) during surgery. The primary clinical endpoint is serum creatinine change from baseline to postoperative day 2, and the primary mechanism endpoint is change in plasma concentrations of F_2_-isoprostanes and isofurans. Secondary endpoints include superoxide production, clinical delirium, myocardial injury, and length of stay. An endothelial function substudy will examine the effects of oxygen treatment and oxidative stress on endothelial function, measured using flow mediated dilation, peripheral arterial tonometry, and wire tension myography of epicardial fat arterioles.

**Discussion:**

The ROCS trial will test the hypothesis that intraoperative physiologic oxygenation decreases oxidative damage and organ injury compared to hyper-oxygenation in patients undergoing cardiac surgery.

**Trial registration:**

ClinicalTrials.gov, ID: NCT02361944. Registered on the 30th of January 2015.

**Electronic supplementary material:**

The online version of this article (doi:10.1186/s13063-017-2021-5) contains supplementary material, which is available to authorized users.

## Background

Each year 500,000 patients undergo cardiac surgery in the USA [1], and up to 30% of these patients develop postoperative acute kidney injury (AKI) [2, 3]. AKI is associated with increased rates of postoperative arrhythmias, wound infections and sepsis, and independently predicts a five-fold increase in death at 30 days [4–6]. We recently demonstrated that intraoperative oxidative damage, quantified by measuring plasma concentrations of F_2_- isoprostanes, independently predicts AKI following cardiac surgery [2]. Excess oxygen exposure (hyperoxia) induces excessive reactive oxygen species (ROS) production in cell-culture and ischemia reperfusion experiments [7–9]. Similarly, hyperoxic reperfusion of ischemic tissues is associated with tissue damage and poor outcomes in some patient populations [10–12].

Anesthesiologists administer supplemental oxygen (fraction of inspired oxygen (FIO_2_) greater than room air (i.e., FIO_2_ > 0.21)) to patients undergoing surgery to avoid hypoxia because prolonged hypoxia leads to organ injury and death. In the majority of patients undergoing surgery, however, supplemental oxygen administration is not required to maintain an arterial hemoglobin oxygen saturation (O_2_ sat) greater than 95%, and with the development of continuous O_2_ sat pulse oximetry, the excess administration of supplemental oxygen may not reduce the incidence of hypoxia. The use of continuous pulse oximetry O_2_ sat monitors and point-of-care arterial blood gas measurement machines provide better assessments of patient oxygenation but have not affected the practice of administering excess supplemental oxygen during surgery. During cardiac surgery anesthesiologists typically ventilate patients with a FIO_2_ of 1.0. In normal conditions, approximately 99% of the oxygen in blood is bound to hemoglobin, and administering oxygen at concentrations higher than those needed to saturate hemoglobin does not increase oxygen content in blood by a clinically important amount. It does, however, increase the partial pressure of oxygen in plasma to super-physiologic levels. These super-physiologic levels may increase the production of ROS [13] which could induce an oxidative stress and lead to organ injury. At the cellular level, oxidative stress causes direct damage to proteins, including deoxyribonucleic acid (DNA) and lipids, resulting in organelle autophagy, cellular apoptosis and necrosis, organ injury and dysfunction, and death [14]. Preclinical studies have also demonstrated that ROS impair endothelial function [15]. The endothelium regulates perfusion. Thus, endothelial dysfunction may contribute to the associations among patient oxygenation, oxidative stress, and organ injury following cardiac surgery.

In other clinical scenarios hyperoxia is harmful. Following cardiac arrest and successful cardiopulmonary resuscitation (CPR); for example, hyper-oxygenation is associated with poor neurologic recovery, coma, and a 44% increase in the odds of death per 100 mmHg change in partial pressure of arterial oxygen (PaO_2_) [16]. This hyperoxia-associated organ injury may be mediated by oxidative stress [11]. In a recent investigation of critically ill patients, the patients randomly assigned to conservative supplemental oxygen administration (goal PaO_2_ 70–100 mmHg and O_2_ sat 94 to 98%) had decreased mortality compared to those assigned to higher oxygen levels/conventional care (PaO_2_ up to 150 mmHg or O_2_ sat 97 to 100%) [17]. In another study, patients suffering myocardial infarction but without hypoxia who were randomly assigned air had reduced infarct size compared to those assigned supplemental oxygen [12]. These findings and the lack of evidence supporting routine administration of hyperoxia support the need to further investigate the effects of conservative supplemental oxygen administration in critically ill patient populations.

We hypothesize that the maintenance of physiologic oxygenation during cardiac surgery, defined as the administration of oxygen concentrations no greater than what is required to maintain arterial hemoglobin saturation at 97%, decreases renal dysfunction and oxidative stress compared to hyper-oxygenation. Specifically, we will test the hypotheses that maintaining physiologic oxygenation (normoxia) during anesthesia for cardiac surgery (1) decreases postoperative kidney (primary clinical endpoint), brain, and myocardial injury, (2) reduces the generation of superoxide radicals in blood, (3) decreases systemic oxidative stress (primary mechanism endpoint), and (4) improves endothelial function compared to hyper-oxygenation (hyperoxia).

## Methods/design

**Fig. 1:**
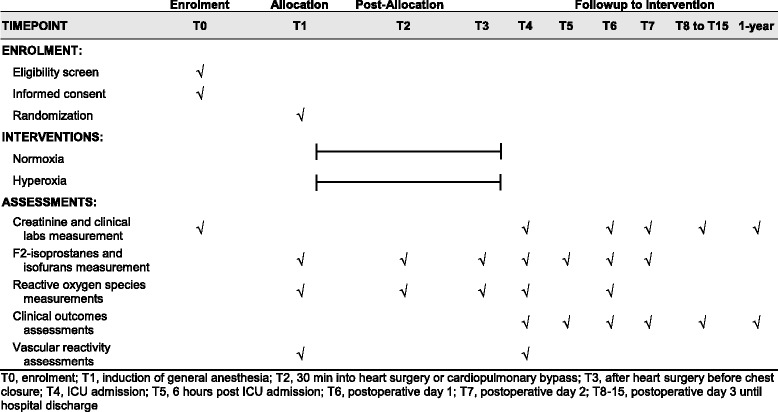
Standard Protocol Items: Recommendations for Interventional Trials (SPIRIT) figure

This is a single-center, randomized, blinded, investigator-initiated clinical trial of 200 patients undergoing cardiac surgery at Vanderbilt University Medical Center (Nashville, TN, USA). We will recruit 220 patients in order to study 200 patients to account for an anticipated 10% of patients who will consent to be studied but will fail exclusion criteria, have surgery cancelled, or withdraw for personal reasons prior to randomization. We will screen and recruit patients prior to surgery, randomly assign them to intraoperative oxygenation treatment, measure proposed mechanism markers prior to, during, and following treatment, and measure clinical outcomes until hospital discharge and again at 1-year follow-up. This protocol conforms to the Standard Protocol Items: Recommendations for Interventional Trials (SPIRIT) guidelines (Fig. 1 and Additional file 1) [18].

### Inclusion criteria

Patients aged 18 years or older undergoing cardiac surgery, defined as surgery on the heart or aorta requiring sternotomy or thoracotomy, will be eligible to participate.

### Exclusion criteria

Patients will be excluded from the trial if they meet any of the following criteria: (1) current acute coronary syndrome defined as ST-elevation myocardial infarction or non-ST-elevation myocardial infarction with troponin leak within 72 h of surgery with or without electrocardiogram changes consistent with myocardial ischemia, (2) home supplemental oxygen use, (3) preoperative supplemental oxygen requirement to maintain arterial O_2_ sat of 92%, (4) right to left intracardiac shunt including atrial septal defect and ventricular septal defect with cor pulmonale, (5) carotid stenosis defined as >50% stenosis of either carotid artery, (6) cardiac surgery that requires intraoperative circulatory arrest such as aortic arch replacement, (7) current use of hemo- or peritoneal dialysis, and (8) pregnancy. Clinical exclusion criteria were created with the goal of avoiding harm to patients. Because the control group receives protocolled management similar to usual care, and patients not participating would receive usual care, we will exclude patients from study if the active treatment might increase harm rather than the control group increasing harm (there are biologic reasons why both active treatment or usual care could increase harm). Specifically, since physiologic oxygenation could increase the risk of hypoxia compared to hyper-oxygenation (usual care), patients with conditions associated with regional tissue hypoxia will be excluded, thereby not exposing these patients to a treatment that could increase risk beyond usual care. This was the logic for excluding patients with acute coronary syndrome and carotid stenosis. Patients who might require significant supplemental oxygen to maintain normoxia will be excluded. This was the logic for excluding patients on supplemental oxygen at home, patients requiring supplemental oxygen to maintain O_2_ sat >92%, and those with right to left intracardiac shunting. Patients who will require circulatory arrest during surgery will be excluded because it will be impossible to provide physiologic oxygenation during extreme iatrogenic hypothermia for those patients if they were randomized to physiologic oxygenation. Patients on dialysis are excluded because of an inability to measure acute kidney injury, the primary endpoint of the trial. Pregnant patients will be excluded because of lack of preliminary safety data related to the intervention on pregnant patients and unborn babies.

### Intervention

Subjects will be randomized to physiologic oxygenation (the minimum FIO_2_ required to maintain an O_2_ sat between 95 and 97% during mechanical ventilation or a PaO_2_ between 80 and 110 mmHg during cardiopulmonary bypass, but no less than that of air (FIO_2_ = 0.21)) or hyper-oxygenation (administration of FIO_2_ = 1.0 during mechanical ventilation and FIO_2_ = 0.8 during cardiopulmonary bypass) during surgery. The intervention is restricted to intraoperative patient management. Regardless of treatment group, all subjects will receive FIO_2_ = 1.0 during induction of anesthesia until tracheal intubation and 6 L/min flow with Ambu® bag ventilation during transport from the operating room to the intensive care unit (ICU) after surgery.

### Oxygen administration and monitoring during surgery

The anesthesiologist will continuously monitor the subject’s arterial O_2_ sat by pulse oximetry, as is standard care. During cardiopulmonary bypass the subject’s perfusionists will continuously monitor the subject’s PaO_2_ with real-time inline co-oximetry, as is standard care.

**Fig. 2:**
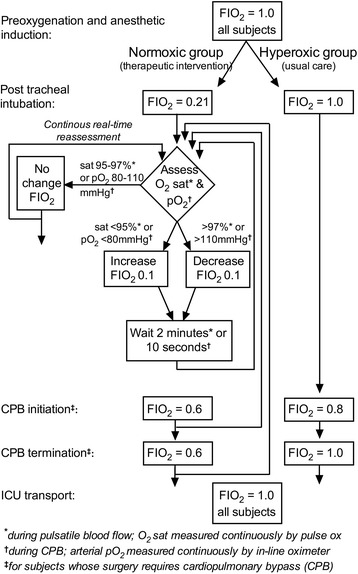
Schematic of oxygen titration protocol

Following induction of anesthesia and tracheal intubation, subjects will be ventilated with a tidal volume of 6–8 mL/kg ideal body weight, a respiratory rate titrated to an end-tidal partial pressure of CO_2_ of 35 cmH_2_O, a positive end-expiratory pressure (PEEP) of 5 mmHg, and a FIO_2_ determined by treatment assignment. A fresh gas flow of at least 2 L/min will be maintained throughout intraoperative mechanical ventilation. The FiO_2_ will be titrated per protocol (Fig. 2).

### Physiologic oxygenation group oxygen titration

Immediately after tracheal intubation the anesthesiologist will decrease the FIO_2_ to 0.21. The anesthesiologist will subsequently increase or decrease the FIO_2_ during lung ventilation in order to maintain arterial O_2_ sat between 95 and 97% but no less than 0.21. If a subject’s O_2_ sat falls below 95% for 1 min, the anesthesiologist will increase the FIO_2_ by 0.1. If the O_2_ sat rises above 97%, the anesthesiologist will decrease the FIO_2_ by 0.1. If the anesthesiologist determines the subject requires more than a 0.1 change in FIO_2_, the adjustment of the FIO_2_ is at the discretion of the anesthesiologist. The anesthesiologist will wait 2 min between changes in FIO_2_ unless the anesthesiologist determines the subject requires more rapid titration of FIO_2_ to maintain the target O_2_ sat of 95–97%.

During initiation of cardiopulmonary bypass, the oxygenator in the cardiopulmonary bypass circuit will be ventilated with FIO_2_ = 0.6 and the gas flow titrated to achieve an arterial blood partial pressure of CO_2_ of 50 mmHg. Immediately following achievement of full cardiopulmonary bypass flow, the FIO_2_ will be decreased to achieve an O_2_ sat between 95 and 97%, and an arterial blood gas sample will be measured to calibrate the inline co-oximeter. Subsequent FIO_2_ titrations during cardiopulmonary bypass will be made using the inline co-oximeter to achieve target PaO_2_ of 80–110 mmHg. If a subject’s PaO_2_ falls below 80 mmHg, the perfusionist will increase the FIO_2_ by 0.1. If the PaO_2_ rises above 110 mmHg, the perfusionist will decrease the FIO_2_ by 0.1. If the anesthesiologist or perfusionist determines the subject requires more than a 0.1 change in FIO_2_, the adjustment of the FIO_2_ is at the discretion of the anesthesiologist and perfusionist. The perfusionist will wait 2 min between changes in FIO_2_ unless the anesthesiologist or perfusionist determines the subject requires more rapid titration of FIO_2_ to maintain a target PaO_2_ of 80–110 mmHg.

### Hyper-oxygenation group oxygen titration

Following verification of tracheal intubation subjects will remain ventilated with a FiO_2_ = 1.0 and will be ventilated with a FiO_2_ = 1.0 throughout all intraoperative mechanical ventilation. During cardiopulmonary bypass, the oxygenator in the cardiopulmonary bypass circuit will be ventilated with a FIO_2_ = 0.8 and gas flow titrated to achieve an arterial blood partial pressure of CO_2_ of 50 mmHg. The FiO_2_ during cardiopulmonary bypass will be increased to maintain a PaO_2_ > 200 mmHg but not decreased below 0.8.

### Transport and ICU ventilation

**Fig. 3:**
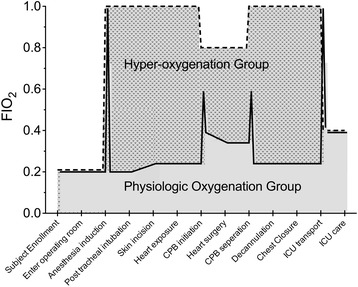
Expected oxygen exposures during surgery (based on preliminary subjects). The hyper-oxygenation curve is shifted 1% left and 1% up to improve discrimination compared to the physiologic oxygenation curve

During transport from the operating room to the ICU following surgery, subjects will be connected to an Ambu® bag and mechanically ventilated with 6 L/min fresh gas flow. Upon arrival in the ICU, subjects will be connected to an ICU ventilator and ventilated with a FIO_2_ = 0.40 or the minimum required to maintain O_2_ sat of 95% if FIO_2_ greater than 0.40 is required, a tidal volume of 8 mL/kg of ideal body weight, a respiratory rate of 12 breaths per min, and PEEP of 8 mmHg, as is usual practice. Expected perioperative oxygen exposures are shown in Fig. 3. Subsequent mechanical ventilation, supplemental oxygen administration, and subject extubation will be at the discretion of the ICU physician.

### Randomization

Randomization will be stratified by chronic kidney disease (CKD), defined as an estimated glomerular filtration rate <60 mL/min/1.73 m^2^, and use of cardiopulmonary bypass during surgery, as these items are risk factors for oxidative stress and kidney injury. For each of the four strata, a computerized random number generator was used to generate a sequence of binary treatment assignments in randomly permuted blocks of size 2 or 4, where block size is selected uniformly at random. The randomization table was uploaded to the centralized automated randomization module in REDCap™. The study statistician wrote the computer program (R; https://www.r-project.org/) that generates the randomization table. However, to preserve the blinding of study personnel, a third-party statistician executed the program and uploaded the resulting tables to the online randomization module. Immediately prior to the subject being brought into the operating room, study personnel access the online randomization module to randomize and learn the treatment assignment for that patient. This type of stratified block randomization ensures a degree of balance both within and across strata, while random selection of block sizes ensures that study personnel cannot predict the treatment assignment of subsequent patients.

### Blinding

Study anesthesiologists and perfusionists will be responsible for protocol-directed administration of physiologic oxygenation or hyper-oxygenation. Therefore, anesthesiologists and perfusionists will be aware of subject treatment assignment. Subjects, surgeons, laboratory technicians, statisticians and ICU staff will remain blinded to treatment group. Preoperative, intraoperative, and postoperative care will remain unaffected by inclusion in this study or by study group assignment aside from oxygenation treatment. Since there is a potential for anesthesiologists to alter the care of patients based on intervention allocation aside from the protocol-directed treatment, we have instituted protocols to guide other aspects of anesthetic care such as mechanical ventilation, vasoactive drug administration, and anesthetic administration, and we will instruct anesthesiologists to not alter their care in any way based on treatment assignment aside from oxygen administration. However, to measure any differences in clinical care between groups that could be a result of oxygenation treatment or the treatment assignment, we also will collect metrics of respiratory, anesthetic, and hemodynamic management, including respiratory rate, tidal volume, PEEP, concentrations of all inhaled and exhaled gases and anesthetics, heart rate, systemic blood pressure, pulmonary artery pressure, central venous pressure, and cardiac output, collected and stored automatically every minute in the perioperative data warehouse; and vasoactive drug administration, fluid administration, and transfusion recorded in the anesthetic record.

We expect subjects randomized to physiologic oxygenation or hyper-oxygenation to have similar vital signs, similar responses to surgery, similar response to medications, and to not warrant any differences in care based on treatment assignment. Surgeons will not be informed of subject assignment, but we will not initiate any procedures, such as concealing oxygen and air flow meters, to actively blind them from assignment. We do not expect any knowledge of subject treatment assignment to affect surgical management of subjects. Objective endpoints, such as laboratory values, are measured by technicians and co-investigators blinded to treatment assignment. Physicians and research staff measure clinical endpoints, such as wound infection and delirium, and are blinded to treatment assignment. The key that lists the treatment of each subject is withheld from investigators, laboratory technicians, and statisticians until the dataset is locked. All preoperative, intraoperative, and postoperative clinical care is administered according to standardized clinical protocol and is not affected by inclusion in this study or by study group assignment.

### Study withdrawal/discontinuation

Subjects who are withdrawn from study participation will cease study-specific oxygen administration and monitoring, and their surgical and postoperative care will proceed as directed by their clinical caregivers.

### Endpoints

The study statistician will calculate the primary endpoint and secondary endpoint results for each patient while blinded to treatment assignment using data documented by the principal investigator (PI), the co-investigators, the research nurse, the research assistant, and the laboratory technicians who are also blinded to treatment assignments. The study statistician will compare endpoints between treatment groups according to the data analysis plan. Institutional protocols for routine cardiac surgery monitoring and clinical laboratory measurements create the clinical data used for clinical endpoint assessments, and the patient’s physicians (intensivist, surgeon, and consultants) make clinical diagnoses used for clinical endpoint assessments using standardized diagnostic criteria (see “Secondary endpoints”).

### Primary endpoints

The primary endpoints are postoperative creatinine change from baseline to postoperative day 2 (primary clinical endpoint), and perioperative plasma F_2_-isoprostane and isofuran concentrations (primary mechanism endpoint). F_2_-isoprostanes and isofurans will be measured at anesthesia induction (T1, baseline), 30 min into heart surgery (T2), immediately following heart surgery but with chest still open (T3), at ICU admission (T4), 6 h after ICU admission (T5), and on the mornings of postoperative days 1 (T6) and 2 (T7) to assess perioperative oxidative damage and will be analyzed using a mixed-effects regression analysis for repeated measures (see “Data analysis plan”).

### Secondary endpoints

Additional markers of AKI include the incidence of AKI using Kidney Disease Improving Global Outcomes (KDIGO) criteria assessed within the first seven postoperative days and urinary markers neutrophil gelatinase-associated lipocalin (NGAL) and insulin-like growth factor binding protein-7 x tissue inhibitor of metalloproteinase-2 (IGFBP-7 x TIMP-2) measured from T1 to T7 [19]. Additional mechanism markers include ROS production using electron spin probes, electron paramagnetic resonance, dihydroethidium, and high-performance liquid chromatography in blood (T1, T3, and T6), myocardium (T3), and epicardial fat (T3); and hypoxia-inducible factor (HIF) signaling endpoints, such as plasma concentrations of adenosine or HIF-1 messenger ribonucleic acid (mRNA), expression in the urine [20, 21]. We will measure markers of mitochondrial function including superoxide production, mitochondrial DNA content, mitochondrial membrane potential, PGC-1a mRNA expression, and mitochondrial respiration in peripheral blood mononuclear cells isolated from blood and atrial tissue.

Secondary clinical endpoints will be assessed from the time of ICU admission up to the time of hospital discharge and include the incidence and duration of delirium, assessed using the Confusion Assessment Method for the ICU (CAM-ICU) twice daily while subjects are in the ICU or at least 3 days postoperatively [22]; myocardial injury, quantified by measuring serum myocardial b fraction of creatine kinase (CK-MB) assessed at T6 and defined as incidence of CKMB greater than 50 ng/mL (10 times the upper limit of normal) and as a continuous endpoint; duration of postoperative mechanical ventilation; development of arrhythmias including atrial fibrillation; dialysis; pneumonia, defined as a positive sputum culture or postoperative pulmonary infiltrate with systemic signs of infection (temperature >38.6 °C or white blood cell count >12.0 × 10^9^/L) and the use of parenteral antibiotics or documentation of the diagnosis by the patient’s physician; wound infection (Center for Disease Control criteria); and ICU length of stay. Pain is assessed on postoperative day 5 and at 1 year, using the Numeric Rating Scale (NRS) 0–10 pain assessment.

One year follow-up data including assessments of cognitive function (Short Blessed Test), activities of daily living, and depression (Center for Epidemiologic Studies-Depression (CES-D) test will be obtained by telephone interview, and we will examine the clinical chart for admissions, events (any renal replacement therapy), and laboratory findings (with particular focus on serum creatinine, estimated glomerular filtration rate, and urine proteinuria) in this period.

### Assessments of oxygenation and protocol adherence

Oxygen administration, tissue oxygenation, and tissue perfusion will be assessed by measuring inspired concentration of oxygen every minute; O_2_ sat each minute during surgery (median ± 95% CI and area-under-the-curve (AUC) <90%), PaO_2_ (median ± 95% CI, AUC >150 mmHg, and AUC <70 mmHg) measured in blood at T1, T2, T3, and T4 and continuously during cardiopulmonary bypass, brain tissue hemoglobin O_2_ sat (median ± 95% CI and AUC <80% baseline) measured every 5 s during surgery, the percentage of FIO_2_ titrations within protocol (protocol adherence), cardiac output using a pulmonary artery catheter placed as part of usual care at T1 and T3, mixed venous hemoglobin O_2_ sat at T1, T3, and T4; and arterial lactate at T1, T2, T3, and T4. To further assess adherence to intervention in the active treatment group, the time elapsed above the protocol-dictated oxygenation targets (O_2_ sat 95–97% during mechanical ventilation and PaO_2_ 80–110 mmHg during cardiopulmonary bypass) and below these targets will be calculated, as well as the AUC above and below these thresholds.

We also collect intraoperative vital signs, electroencephalographic activity, medication use, fluid input, urine output, and clinical laboratory tests to phenotype patients.

### Effects on endothelial function substudy

The primary endpoint of the endothelial function substudy is vascular reactivity measured using brachial artery flow-mediated dilation (FMD, primary endpoint of the substudy) and digital peripheral arterial tonometry (PAT) after induction of general anesthesia and again at ICU admission. We will directly measure vascular reactivity in blood vessels dissected from epicardial fat at T3 in a subset of subjects chosen at random. We will quantify markers of endothelial activation in plasma including plasminogen activator inhibitor-1 (PAI-1) and E-selectin. Associations between vascular reactivity, endothelial activation markers, ROS production, oxidative stress, and clinical outcomes will be examined.

### Safety endpoints

Safety endpoints focus on any risk of hypoxia between study groups and will include postoperative myocardial infarction (defined by a new Q wave on the electrocardiogram), rates of reintubation, transient ischemic attack (defined as new deficit on neurologic exam that abates within 72 h), stroke (defined as new deficit on neurologic exam and confirmed with radiologic evidence), and death. Safety endpoints will be assessed until hospital discharge.

### Urine, blood, and tissue use

Following the placement of the arterial line and Foley catheter, 20 mL blood and 10 mL urine will be collected at the induction of anesthesia (baseline, T1). We also collect 15 mL blood and 10 mL urine during cardiopulmonary bypass or off-pump coronary artery bypass (T2), following cardiopulmonary bypass or off-pump coronary artery bypass (T3), at ICU admission (T4), 6 h after ICU admission (T5), and on postoperative day 1 (T6) and day 2 (T7). In total we collect 110 mL blood and 70 mL urine. When possible, after discontinuation of cardiopulmonary bypass, 1–2 g of mediastinal fat dissected as part of the surgery and 1–2 g of redundant atrial tissue at the site of venous cannula insertion will be collected for measurement of atrial myocardial function, inflammation, amyloid, oxidative stress, and arteriolar vascular reactivity. Typically, this tissue is discarded, but instead we will harvest this tissue at the end of surgery as we have done previously [23]. Biologic samples will be securely stored and frozen at −80 °C.

### Data and Safety Monitoring Board (DSMB)

The DSMB will provide objective review of the treatment results as they relate to human safety and data quality. The DSMB will receive report on the progress, adverse events, safety endpoints, and clinical outcomes at each interim analysis from the PI and the statistician. Interim analyses will occur after 20, 100, and 200 subjects have been studied. The DSMB will evaluate subject recruitment, the documentation of subject data, and the collection and reporting of adverse events, serious adverse events, and safety endpoints (stroke, transient ischemic attack, Q-wave myocardial infarction) and conditional power analysis. The conditional power analysis will assess the likelihood of rejecting the null hypotheses at the study conclusion (*N* = 200), assuming that the effect of intervention is as large or larger than the estimated effect at the second interim analysis (*N* = 100). To do so, the study statistician will compare mock-unblinded data (A versus B, where it remains unknown if A or B is normoxia or hyperoxia). If the conditional power is less than the original target (80%), the DSMB will consider recommending an increase in the study sample size to achieve the target power. If this recommendation is made, the investigators will apply to the Vanderbilt University Medical Center Institutional Review Board (IRB) for approval for additional subject recruitment. There will be no possibility of ending the trial early due to futility or efficacy. All investigators and research personnel (other than the study statistician at the second interim analysis) will remain blinded to treatment assignment during all interim analyses. At either planned interim analysis the DSMB may recommend to continue enrollment and subject study or suspend enrollment and subject study due to safety concerns secondary to the study intervention.

### Adverse events

Adverse events (AE) are monitored by the PI and the co-investigators. AEs are submitted to the IRB as per IRB regulation. Specifically, any severe AE determined to be possibly, probably, or definitely related to study participation is reported to the DSMB and the IRB within ten working days of the PI’s notification of the event. Summary reports of any previously unreported severe AEs will be reported to the IRB annually and to the DSMB at interim analyses and as requested. These reports will contain the number of AEs, a description of each AE, the category of each AE, and the relationship of each AE to study participation.

## Statistical analysis plan

The primary objective of the analysis will be to compare the effect of intraoperative oxygenation on oxidative damage and organ injury. The primary endpoints will be change in serum creatinine from baseline (defined as the most recent creatinine measured prior to surgery) to postoperative day 2 (primary clinical endpoint) and F_2_-isoprostanes/isofurans measured in plasma (primary mechanism endpoint). Primary endpoints of the endothelial function substudy will be change in vascular reactivity tests before and after treatment and associations with plasma F_2_-isoprostanes/isofurans.

### Sample size calculations

We calculated the subject sample size based on data from the Vanderbilt Statin AKI Cardiac Surgery randomized clinical trial [24]. Data from this study indicate that mean serum creatinine concentrations increase by 0.08 ± 0.37 mg/dl from baseline to postoperative day 2. We have powered the present randomized clinical trial conservatively, particularly since consensus criteria for AKI diagnosis define AKI as a 0.3-mg/dl rise in serum creatinine from baseline to postoperative day 2 [25]. With 200 subjects, we will have 80% power to detect a 0.15 ± 0.375-mg/dL difference in serum creatinine between groups, with a type I error rate of 5%. In addition, with 200 subjects we will have 80% power to detect a 20 ± 50-pg/mL difference in plasma F_2_-isoprostanes between groups. To account for a potential dropout rate of 10%, we plan to recruit 220 subjects.

### Data analysis plan

Standard graphing and screening techniques will be used to evaluate data quality. Data include patient demographics, baseline and intraoperative clinical characteristics, treatment toxicity and clinical outcomes. Summary statistics of study arms for both numerical and categorical variables will be provided to describe the study sample. Continuous variables will be summarized with the 50th (10th, 25th, 75th, 90th) percentiles, mean and standard deviation, and categorical variables will be summarized with the counts and percentages. For outcomes measured at a single time point, comparisons across randomization groups will be implemented using the Fisher’s exact test or Pearson chi-square test for categorical variables, and the Wilcoxon rank sum test or Student’s *t* test for quantitative outcomes, as appropriate. The Clopper-Pearson and Wald methods will be used to construct 95% confidence intervals for the between group effects.

Change in serum creatinine from baseline to postoperative day 2 (primary clinical endpoint) will be compared between normoxia and hyperoxia study groups using the Wilcoxon rank sum test. Plasma concentrations of F_2_-isoprostanes and isofurans (primary mechanism endpoint) will be compared between normoxia and hyperoxia study groups using a mixed-effects regression analysis, adjusting study time point as a categorical variable (i.e., study phase). Analysis of the effects of treatment on change in creatinine and on oxidative stress will be repeated separately within randomization strata (with and without CKD and with and without use of cardiopulmonary bypass).

Despite prospective randomization, a propensity-score adjusted analysis will be considered to adjust for markedly imbalanced allocation. The propensity score will be derived from a binary logistic regression of randomized treatment assignment on risk factors for AKI and oxidative stress. A natural cubic spline method will be used to evaluate the possibility of nonlinear associations between quantitative predictors and the odds of treatment allocation.

We will evaluate treatment effect on additional markers of AKI (AKI incidence using KDIGO criteria, urine concentrations of NGAL and IGFBP-7/TIMP2, use of dialysis), delirium incidence and duration (diagnosed with CAM-ICU), and myocardial damage (quantified by measuring CK-MB on the morning of the day after surgery) as secondary analyses.

To assess the effect of treatment on oxygenation and perfusion we will compare O_2_ sat, PaO_2_, cerebral oxygenation, muscle oxygenation, mixed venous hemoglobin saturation, cardiac output, and arterial lactate concentrations between normoxia and hyperoxia groups using mixed-effects regression analysis as described above for plasma concentrations of F_2_-isoprostanes and isofurans.

We will also compare differences in ROS production, mitochondrial function, atrial fibrillation, stroke, pneumonia, wound infection, time-to-extubation, duration of ICU stay, and in-hospital mortality as well as pain scales, depression data, and neurocognitive function between normoxia and hyperoxia groups as secondary endpoints. We will compare safety endpoints myocardial infarction, transient ischemic attack, and stroke between treatment groups. Similar to the primary analysis, we will implement logistic, proportional odds and linear regression analyses for binary, ordinal or highly skewed, and continuous outcomes, respectively, using a propensity score adjusted approach, if warranted. Potential impactful covariates included in these models will be specific to each outcome.

To test the hypothesis that intraoperative physiologic oxygenation improves postoperative endothelial function (endothelial function substudy) we will compare change from baseline in FMD (primary endpoint of endothelial function substudy), reactive hyperemia index (RHI) calculated from PAT, PAI-1, and E-selectin concentrations, and the amplitude of endothelium-dependent vasodilation between randomized subject groups. The change from baseline in FMD, RHI, PAI-1, and E-selectin and the amplitude of endothelium-dependent vasodilation will be compared between oxygenation groups using Student’s *t* test or the Mann-Whitney *U* test. To test the hypothesis that endothelial dysfunction correlates with increased oxidative stress during cardiac surgery, we will compare perioperative concentrations of F_2_-isoprostanes and isofurans to endothelial function assessments and plasma markers of endothelial activation at ICU admission using linear regression and adjust for baseline measurements of endothelial function in addition to confounders such as randomized treatment group, blood transfusion, and duration of cardiopulmonary bypass. To test the hypothesis that perioperative endothelial dysfunction is associated with organ injury, we will compare baseline, postoperative, and change from baseline FMD, RHI, PAI-1, and E-selectin and the amplitude of endothelium-dependent vasodilation to markers of AKI, delirium, myocardial injury, and additional secondary endpoints using logistic, linear, or proportional odds regression as appropriate.

Every effort will be made to avoid missing data. Any methods of missing data imputation (e.g., multiple imputation using the chained-equations method) will be used cautiously as sensitivity analyses; and the analysis of results with imputation would be interpreted in the context of corroboration with the analyses without imputation. The primary analysis will be intention-to-treat, meaning that all patients will be analyzed as being members of the assigned group regardless of treatment received. Before data unblinding, we will examine any protocol deviations and identify a list of protocol violators. In addition to the intention-to-treat analysis, a secondary per-protocol analysis will be conducted.

Specific inferences on the effects of interest will be made by reporting point estimates and 95% confidence intervals. Hypotheses will be tested at a two-sided significance level of *α* = 0.05. This data analysis plan will be carried out using the statistical analysis package R (R Development Core Team). Results will be published after completion of the trial and after data analysis is complete.

Data will be stored, curated, and secured in the Vanderbilt University REDCap™ system [26]. Access is limited to study personnel and statisticians.

## Discussion

Hyper-oxygenation during cardiac surgery is a common practice, but hyper-oxygenation might increase organ injury by increasing the production of ROS and increasing oxidative damage. The ROCS trial will test the hypothesis that physiologic oxygenation during surgery will decrease the production of ROS, oxidative damage, and organ injury compared to hyper-oxygenation.

Two recent studies have been conducted to test similar hypotheses. First, the SO-COOL study randomly assigned cardiac surgery patients to avoidance of hyperoxemia versus usual care during cardiopulmonary bypass at two hospitals in New Zealand and Australia [27]. Here, McGuinness et al. reported no difference in AKI measured using creatinine and urine output KDIGO criteria or in other secondary clinical markers. The study protocol did not limit hyperoxia prior to, or following, cardiopulmonary bypass, and during these periods patients were hyper-oxygenated. Following cardiopulmonary bypass pulsatile flow is restored, and ischemic tissues may be reperfused. In experimental models markers of oxidative damage surge following reperfusion [7–9, 28]. Markers of oxidative stress also surge after cardiopulmonary bypass [29, 30]. In addition, measurements of perioperative oxidative stress were not reported, thus it is unknown if the intervention decreased the putative mechanism for organ injury related to hyper-oxygenation.

Second, the Oxygen-ICU Randomized Clinical Trial randomly assigned critically ill patients in a medical-surgery ICU to conservative oxygenation (target PaO_2_ 70–100 mmHg or O_2_ sat 94–98%) or standard ICU practice [17]. Although the median PaO_2_ in the conservative oxygenation group was only 15 mmHg lower than the median PaO_2_ in the control group, the conservative oxygenation group had a shorter duration of mechanical ventilation and lower rates of death, shock, liver failure, and bacteremia. Renal failure was similar between groups. This study was terminated early due to difficulty with enrollment.

Dissimilar from prior studies, the ROCS trial will randomize cardiac surgery patients to physiologic oxygenation or hyper-oxygenation throughout the entire operative period and will measure the putative mechanisms involved, namely oxidative stress and endothelial function, in addition to clinical outcomes.

Limitations of the ROCS trial include an inability to blind anesthesiologists to intervention assignment. This is not possible since anesthesiologists are required to implement the oxygen titration protocol, but it creates a risk for study groups to be treated differently, masking the effect of intervention. Similarly, while cardiac surgeons will not be informed of patient treatment assignment and will be informed that they cannot ascertain treatment assignment, surgeons could examine oxygen flow meters on the anesthesia machine to ascertain treatment. If this were to happen, we do not expect that it would alter surgical technique or management or affect any outcome assessments. Another limitation is the use of serum creatinine as a marker of renal injury in the primary analysis. Serum concentrations of creatinine may be affected by processes aside from renal injury, and the use of serum creatinine change to diagnose AKI may limit accurate measurement of renal injury. However, current consensus guidelines designate change in serum creatinine as the primary metric for AKI diagnosis [19]. We will also measure urine concentrations of AKI biomarkers and the need for renal replacement therapy to phenotype AKI. And finally, the study is limited by a poor ability to assess oxygen tensions in organ tissues of interest, specifically within tubule cells in the kidney and neurons in the brain. We will, however, use near-infrared spectroscopy to measure brain tissue hemoglobin O_2_ sat, a metric that we and others have correlated with acute brain injury following cardiac surgery [31].

The high incidence of AKI, delirium, arrhythmia, and other organ injuries after cardiac surgery and the associations between these organ injuries and oxidative damage warrant the investigation of intraoperative physiologic oxygenation as a means to reduce organ injury and patient morbidity. The ROCS trial will provide insights into mechanisms of perioperative oxidative stress and organ injury and potentially demonstrate that maintenance of physiologic oxygenation during surgery improves the care of cardiac surgery patients compared to the hyper-oxygenation that patients typically receive.

## Trial status

ROCS trial subject recruitment started in April 2016 and 68 subjects have been enrolled. The study’s Data Safety Monitoring Board examined safety endpoints in the first 20 subjects on the 17th of October 2016 and recommended continuation of the trial with no changes to the protocol or consent. Target enrollment will be achieved in 2019.

## Additional file

Additional file 1: SPIRIT 2013 Checklist. (PDF 877 kb)

## Abbreviations

AE: Adverse event
AKI: Acute kidney injury
CAM-ICU: Confusion Assessment Method for the ICU
CKD: Chronic kidney disease
FIO_2_: Fraction of inspired oxygen
FMD: Flow-mediated dilation
IGFBP-7/TIMP2: Insulin-like growth factor binding protein-7/tissue inhibitor of metalloproteinase-2
IRB: Institutional Review Board
KDIGO: Kidney Disease Improving Global Outcomes
NGAL: Neutrophil gelatinase-associated lipocalin
O_2_ sat: Arterial hemoglobin oxygen saturation
PAI-1: Plasminogen activator inhibitor-1
PaO_2_: Partial pressure of arterial oxygen
PAT: Peripheral arterial tonometry
RHI: Reactive Hyperemia Index
ROCS: Risk of Oxygen during Cardiac Surgery
ROS: Reactive oxygen species
